# Gelatinous Marrow Transformation Associated with Imatinib: Case Report and Literature Review

**DOI:** 10.1155/2017/1950724

**Published:** 2017-01-04

**Authors:** E. Chang, G. Rivero, B. Jiang, S. Yellapragada, P. Thiagarajan

**Affiliations:** ^1^Department of Hematology and Oncology, Michael E. DeBakey Veterans Affairs Medical Center, Houston, TX 77030, USA; ^2^Baylor College of Medicine, 1 Baylor Plaza, Houston, TX 77030, USA; ^3^Endocrinology, Michael E. DeBakey Veterans Affairs Medical Center, Houston, TX 77030, USA; ^4^Department of Pathology, Michael E. DeBakey Veterans Affairs Medical Center, Houston, TX 77030, USA

## Abstract

Gelatinous marrow transformation (GMT) is a rare condition observed in severe illness or malnutrition, in which the bone marrow contains amorphous “gelatinous” extracellular material, and histopathology demonstrates varied degrees of fat cell atrophy and loss of hematopoietic elements. An association of GMT with imatinib use in chronic myeloid leukemia (CML) has been reported recently. The objective of this study is to describe a case of GMT associated with imatinib use and review the existing similar cases in the literature to identify epidemiological patterns and potential imatinib-induced mechanisms leading to gelatinous conversion.

## 1. Introduction

Gelatinous marrow transformation (GMT) is a condition of poorly understood pathophysiology observed in cachexia or severe illness, such as sepsis or widespread malignancy. It is characterized by amorphous extracellular material which appears gelatinous. Histopathology is striking for Alcian-blue-staining eosinophilic substances, fat cell atrophy, and uneven hypoplasia. An association of GMT with use of tyrosine kinase inhibitors (TKIs), such as imatinib, has been reported. However, the underlying mechanisms resulting in drug-induced hematopoietic gelatinous conversion are unknown. The objective of this paper is to describe a case of GMT following the use of imatinib, review previous cases of GMT associated with imatinib therapy, and highlight epidemiologic, clinical, cytogenetic, and molecular features associated with the complication.

### 1.1. Case Presentation

A 78-year-old man presented to our institution in 2011 with* BCR-ABL1 *positive CML diagnosed in 2004. He had been treated with imatinib. For the first year, he received 200–300 mg orally daily, due to thrombocytopenia. Hemoglobin (Hb) remained at 11 g/dL [normal range, 12–18 g/dL] and white blood cell (WBC) count normalized from 50 × 10^9^/L to 5 × 10^9^/L [normal range, 4–11 × 10^9^/L]. Platelet count fluctuated around 50–100 × 10^9^/L [normal range, 150–450 × 10^9^/L.]. After 13 months, imatinib was held due to skin complications. Cytogenetic and molecular remission data were not available from 2005 to 2011. Dementia associated with confusion and memory deficits became apparent in early 2011. Progressive weight loss of 18 kg resulted in body mass index (BMI) and weight of 18 and 54 kg, respectively. At the end of 2011, eight months later, his weight was stable; however, his complete blood count (CBC) showed a WBC, hemoglobin (Hb), red cell distribution width (RDW), and platelet count of 15 × 10^9^/L, 10 g/dL, 14% [normal range, 11.6–14.6%], and 160 × 10^9^/L, respectively. ANC and ALC were 9 × 10^9^/L and 4 × 10^9^/L [normal ranges, 1.5–8.0 × 10^9^/L and 1.3–3.5 × 10^9^/L, respectively].

**Figure 1:**
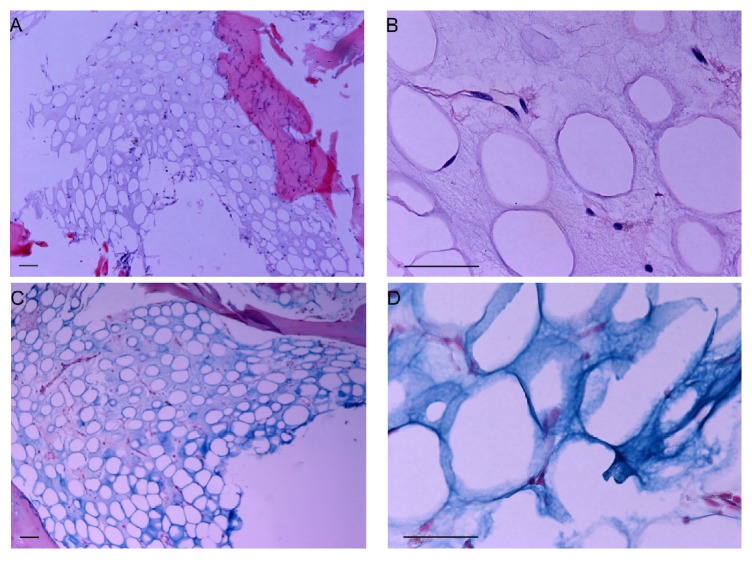
Gelatinous transformation in the bone marrow. Trephine biopsy of the bone marrow specimen stained with hematoxylin and eosin (A and B); Alcian blue (C and D). Scale bars are 50 *μ*m. Images were obtained at 100x (A and C) and 500x (B and D).

A baseline bone marrow biopsy showed trilineage hematopoiesis with a cellularity of 60%, a myeloerythroid ratio of 8 : 1, and the Philadelphia chromosome, consistent with CML. BCR-ABL : ABL PCR ratio was 5%. He restarted imatinib at 400 mg orally daily in late 2011. After two months of treatment, BCR-ABL : ABL ratio was 0.8%. Over the next 8–10 months, he developed anemia and thrombocytopenia (Hb decreased from 10 down to 8 g/dL and platelet count from 150 to 40 × 10^9^/L). His weight had decreased by 2 kg, albumin was 3.5 g/dL, and HIV status was negative. His repeat bone marrow biopsy showed markedly decreased cellularity and amorphous gelatinous substances staining positive with Alcian blue (Figure 1(A)). BCR-ABL : ABL ratio had increased to 14%, consistent with progression. TKI was stopped given suspicion for hematopoietic failure linked to GMT. Due to progressive dementia, his family decided against therapy. His CBC showed a WBC, Hb, and platelet count of 20 × 10^9^/L, 7.3 g/dL, 136 × 10^9^/L. He was transferred to hospice care.

## 2. Discussion and Review of Imatinib-Associated Cases

GMT, also known as “starvation bone marrow,” is a rare diagnosis among bone marrow biopsies. Although the morphologic findings were noted as early as 1900 [1], understanding of its pathophysiology and clinical significance is still limited. Most cases are associated with weight loss or cachexia. To date, the analysis by Böhm represents the largest report, in which 155 cases were found from more than 80,000 marrow specimens, demonstrating a low frequency of 0.2% [2].

While the association between cachexia and GMT is well known, the link between imatinib and GMT is not yet well-defined. In addition to the 155 cases described by Böhm in which none were associated with CML or imatinib, a second study including 65 GMT cases failed to demonstrate CML or imatinib as potential culprits [3]. Additionally, a retrospective review spanning records over 1.5 years found that, among 683 patients who were treated with imatinib, 60 patients (9%) developed cytopenias and only one of those cases was diagnosed with GMT [4].

### 2.1. Cohort Analysis

To our knowledge, six cases of GMT associated with imatinib therapy have been reported (Table 1) [4–9]. All patients had CML. Median duration of therapy was 12 months (range, 4–36 months) prior to GMT diagnosis. Median dose was 400 mg (range, 400–600 mg). Among the 4 patients with low counts, the median time to onset of cytopenias was 8 months (range, 4–36 months). Imatinib was continued in 3 of the patients; switched to a different TKI in 1 patient; and stopped in 2 patients (1 patient proceeded to transplant, and our patient had a change in goals of care). Whenever possible, authors were contacted to complete clinicopathological data. However, BMI at onset of therapy and weight trends were not always available to suggest weight loss as etiology. In at least 3 of the cases, weight loss and malnutrition were not evident. Although the clinical significance of GMT in CML patients treated with imatinib is unknown, it does not always correlate with treatment failure or progression. This appears to be particularly true in younger patients, such as in the first four cases (4/7, 57%) in Table 1. These four cases had documented cytogenetic or major molecular remission. In contrast, 2/7 (29%) of patients' (our case and one from our cohort analysis) cytogenetics or molecular studies suggested refractory CML. Median age for patients with cytogenetic/molecular remission versus those with relapsed disease was 60 years and 72 years, respectively, *p* = .036. Evidence for clonal CML hematopoiesis suggests that GMT could be mechanistically associated with CML progression in older patients.

## 3. Proposed Mechanisms

The mechanistic link between GMT and TKI treatment remains incompletely understood, but it appears to begin within the complex stromal and hematopoietic interaction. Though these interactions are still poorly characterized, in vitro models show that direct stromal cell-blood cell contact, extracellular marrow matrix, and cytokine synthesis are all important to the hematopoietic stem cell (HSC) niche [10]. The deposition of gelatinous substances and catabolism of adipose has been recognized to reduce hematopoietic marrow potential. Histochemical studies indicate that the gelatinous substance is a mucopolysaccharide, and use of hyaluronidase demonstrates that it is specifically hyaluronic acid [11]. Since hyaluronic acid limits the movement of large proteins, its excess may interfere with cell signaling balance within the marrow microenvironment. Furthermore, fat cell atrophy, in animals, causes even more unexplained stimulation of the synthesis of sulfated glycosaminoglycans and hyaluronic acid [12]. Human studies indicate that, in most patients, marrow adiposity increases in lean states and states of caloric restriction [12]. Starvation and TKI therapy may induce similar pathologic responses within the HSC niche. Among other mechanisms, imatinib alters dynamics of marrow connective tissue. It inhibits the growth not only of cells with constitutively active tyrosine kinases but also of benign mesenchymal stem cells in vitro by blocking the tyrosine kinase activity of* c-Kit* and platelet derived growth factor receptor *β* (PDGFR *β*) [13]. Mesenchymal stem cells differentiate into osteoblasts and adipocytes. The inhibitory effect on PDGFR *β* causes skewed adipogenic differentiation over osteogenic, mimicking starvation [13]. Additionally, in calorically restricted mice, starvation increases marrow adiposity and decreases bone density [14], also consistent with the known risk of osteoporosis in patients with anorexia nervosa and the early cytohistologic changes observed in these patients during the progression towards GMT [15]. One of the mechanisms by which starvation slows energy-intensive biosynthetic processes is via inhibition of mammalian target of rapamycin (mTor), which normally signal downstream from* c-Kit *[16]. The molecular and pathologic changes in the marrow microenvironment associated with TKI-induced* c-Kit *blockade may therefore be similar to those seen in starvation.

One limitation for our study is that our patient experienced weight loss of 25%, attributed to dementia. Cachexia and weight loss are both common causes of GMT. However, his bone marrow biopsy after the weight loss, but prior to restarting imatinib, did not show GMT. In contrast, his last biopsy demonstrating GMT was not preceded by significant weight loss, suggesting that malnutrition was not a significant contributor.

The distinction between imatinib-associated GMT and other forms of GMT is worthwhile because, clinically, the former has been at least observationally noted to be more acute in onset and transient in comparison to cachexia, infection, or malignancy-associated GMT [5]. Although distinguishing TKI-induced GMT from CML-induced GMT may not always be possible, in many reported cases of younger patients, a major molecular response and favorable clinical outcomes were achieved as GMT occurred, suggesting that GMT resulted directly from treatment.

Our analyzed cohort appears to suggest two distinct clinical phenotypes: firstly, older patients who develop GMT in less than 6 months and have severe, refractory CML and, secondly, younger patients with GMT who sustain cytogenetic and molecular response. It is still unclear how TKI, advanced age, malnutrition, and CML relapse compound the risk for GMT. However, one hypothesis is that preceding weight loss primes bone marrow to undergo GMT. If malnourishment facilitates GMT in CML patients receiving TKI, then nutritional support would minimize this complication.

Considerations for future investigation should include whether imatinib-associated GMT is reversible and whether newer TKIs have a similar potential for GMT. A larger cohort of CML patients exhibiting GMT could facilitate understanding for this entity.

In conclusion, clinicians should be aware of GMT as a possible sequela of imatinib therapy, especially as the use of imatinib increases. The reported case and cohort analysis add, for the first time, potential clinical and molecular understanding in GMT vulnerability. The observation that imatinib facilitates GMT suggests that investigating molecular alterations induced by the drug could assist in not only selection of alternative therapies but also interventions aimed at reversing the complication.

## Figures and Tables

**Table 1:** Clinical characteristics of patients with gelatinous marrow transformation and CML treated with imatinib.

Case	Author	Age, gender	Cytopenias	Dose and duration of therapy	Status of disease	Outcome	Comorbidities
1	Hong et al. [6]	57, male	Anemia, leukopenia	1 year (400 mg daily)	MMR 1 year earlier^1^	Lowered the dose to 300 mg due to bicytopenia	None: no weight loss or malnutrition
2	Hong et al. [6]	23, female	None	22 months (400 mg daily)	MMR	Stopped imatinib (per patient request), underwent allogeneic transplant	None: no weight loss or malnutrition
3	Agrawal et al. [7]	44, male	None	11 months (400–600 mg daily)	CCyR, MMR	Remains in MMR after 7 years of imatinib	None: no weight loss, malnutrition, or dementia
4	Srinivas et al. [8]	60, male	Pancytopenia	3 years (400 mg daily)	CHR; CCyR 1 year earlier	At 4-month follow-up, leukopenia and mild thrombocytopenia persisted, and imatinib therapy continued	Transitional cell carcinoma of bladder, sepsis, disseminated intravascular coagulation, renal failure
5	Ram et al. [5] and Thakral et al. [9]	Unknown	Grade 2 cytopenias	Unknown	Unknown	Unknown	Unknown
6	Seaman et al. [10]	67, male	Pancytopenia	4 months	No cytogenetic response	Imatinib discontinued, replaced w/nilotinib Subsequent marrows showed restitution of cellularity, but patient continued to have residual disease by RT-PCR or FISH at 8 months	Unknown
7	Our case	78, male	Anemia, thrombocytopenia	Intermittent for years, then 8 months at 400 mg daily	BCR-ABL increasing	Remained off TKIs CML progressed but patient died of other causes	Dementia, diabetes mellitus No weight loss or malnutrition

^1^At time of bone marrow biopsy, the patient was at least in CHR.

CHR = complete hematological remission; MMR = major molecular response; CCyR = complete cytogenetic response.
